# Testosterone Replacement Therapy in Men Aged 50 and Above: A Narrative Review of Evidence-Based Benefits, Safety Considerations, and Clinical Recommendations

**DOI:** 10.7759/cureus.92538

**Published:** 2025-09-17

**Authors:** Luis M Canal de Velasco, José Emiliano González Flores, Jose L Morales Arteaga

**Affiliations:** 1 Medical Education and Simulation, Panamerican University, Mexico City, MEX; 2 Medical Education and Simulation, Tecnológico de Monterrey Campus Ciudad de Mexico, Mexico City, MEX; 3 Cardiology, Panamerican University, Mexico City, MEX

**Keywords:** androgen deficiency, clinical guidelines, late-onset hypogonadism, older men, replacement therapy, testosterone

## Abstract

Testosterone replacement therapy (TRT) is a cornerstone in the management of late-onset hypogonadism in men aged 50 years and above, a condition characterized by progressive declines in serum testosterone, impaired sexual and physical function, metabolic disturbances, and reduced quality of life. Although historical concerns have centered on cardiovascular safety and prostate health, contemporary high-quality evidence and clinical guidelines increasingly support TRT when appropriately prescribed and monitored. To synthesize and critically appraise the evidence regarding the benefits, safety profile, and clinical recommendations for TRT in men aged ≥50 years, a comprehensive search of PubMed/MEDLINE, Embase, and the Cochrane Library (January 2000-July 2025) was conducted, identifying randomized controlled trials, cohort studies, systematic reviews, and meta-analyses assessing TRT in this population. Eligible studies reported outcomes in sexual, musculoskeletal, metabolic, cardiovascular, cognitive, and quality-of-life domains, as well as safety endpoints. TRT consistently improved sexual desire, erectile function, lean body mass, bone mineral density, insulin sensitivity, and vitality, with most benefits observed in men with baseline testosterone levels <300 ng/dL and target levels maintained at 500-800 ng/dL. Safety data indicate no increased risk of major adverse cardiovascular events or prostate cancer when guidelines are followed, though erythrocytosis remains the most common dose-related adverse effect. Effect sizes varied according to baseline androgen status, formulation, treatment duration, and comorbidities. TRT in men aged ≥50 with confirmed hypogonadism offers significant, multi-system benefits with a favorable safety profile under structured monitoring. Individualized treatment strategies and further large-scale, long-term trials are needed to refine patient selection, optimize outcomes, and address remaining knowledge gaps.

## Introduction and background

Testosterone levels in men gradually decline with age, typically beginning in the fourth decade of life and accelerating thereafter. This process, often described as late-onset hypogonadism (LOH) or age-related testosterone deficiency, remains debated in terms of its clinical significance, as it may be influenced by factors such as obesity, metabolic syndrome, type 2 diabetes, and chronic illness [1,2]. Epidemiological studies, including the European Male Ageing Study, report that 20-30% of men aged 50 years and above present serum testosterone concentrations below commonly applied reference ranges; however, such findings vary depending on the study population and diagnostic criteria, and do not by themselves establish hypogonadism [1,3-6].

The clinical manifestations of testosterone deficiency are diverse and include reduced libido (considered the principal correlate of low testosterone), erectile dysfunction (often multifactorial), decreased muscle mass and strength, increased fat mass, low bone density, fatigue, and mood disturbances. These symptoms can collectively contribute to impaired quality of life [3,4]. Testosterone replacement therapy (TRT) has therefore emerged as a therapeutic strategy to restore hormone levels and alleviate symptoms, with some evidence suggesting potential benefits in long-term health outcomes, though results remain mixed and population-dependent [3-6].

Despite historical concerns regarding cardiovascular safety and prostate health, contemporary evidence from randomized controlled trials (RCTs), longitudinal cohort studies, and meta-analyses increasingly supports the use of TRT in carefully selected men aged 50 years and older [2-4]. Current guidelines from the Endocrine Society and the American Urological Association recommend considering TRT in symptomatic men with consistently low morning testosterone levels confirmed by reliable assays [3,4]. Furthermore, accumulating data suggest benefits beyond sexual function, including improvements in bone mineral density, muscle mass, insulin sensitivity, and vitality, although results remain inconsistent across studies [1,4].

Given the growing clinical use of TRT and persistent uncertainties regarding its long-term impact, a focused appraisal of the literature in this age group is warranted. This narrative review aims to synthesize and critically evaluate the available evidence on the benefits, risks, and monitoring parameters of TRT in men aged 50 years and above, with the goal of providing clinicians with a balanced, evidence-based perspective to inform safe and effective clinical decision-making.

## Review

Methods

A literature search was performed in PubMed/MEDLINE, Embase, and the Cochrane Library from January 2000 to July 2025 to identify relevant studies on the clinical benefits, safety, and recommendations for TRT in men aged 50 years and above. Search terms included combinations of "testosterone replacement therapy", "androgen therapy", "late-onset hypogonadism", "older men", "elderly men", "benefits", "safety", and "guidelines".

Studies considered for inclusion involved men aged ≥50 years with confirmed testosterone deficiency or LOH and reported outcomes related to clinical benefits, such as sexual function, muscle mass, bone mineral density, cognition, quality of life, or metabolic parameters, and/or safety, including cardiovascular health, prostate status, and hematological effects. RCTs, cohort studies, systematic reviews, and meta-analyses were reviewed.

Articles were excluded if they involved animal models, were case reports, were conference abstracts without peer-reviewed publication, included participants younger than 50 years without subgroup analysis, or were not in English. Priority was given to high-quality studies and recent clinical guidelines from the Endocrine Society, American Urological Association, and European Academy of Andrology to ensure an evidence-based and clinically relevant synthesis.

Central body

Sexual Function and Libido

Testosterone is a key regulator of sexual desire, erectile capacity, and overall sexual satisfaction in men [5,6]. Age-related declines in serum testosterone, particularly when total testosterone falls below 300 ng/dL (10.4 nmol/L), are strongly associated with reduced libido, increased incidence of erectile dysfunction, and decreased frequency of sexual activity [7,8]. These symptoms tend to be more pronounced in men with levels under 230 ng/dL (8 nmol/L), where androgen-sensitive tissues are more significantly affected [8].

Multiple RCTs and meta-analyses have shown that TRT in hypogonadal men aged 50 years and older leads to measurable and clinically meaningful improvements in sexual desire, frequency of intercourse, and erectile function [4,9,10]. In one RCT (mean baseline testosterone ~234 ng/dL), men treated with transdermal testosterone gel achieved mean on-treatment levels of ~500-700 ng/dL, with significant improvements in sexual activity, desire, and erectile function over 12 months [4]. Benefits were typically noticeable within the first three months of therapy and were sustained throughout the treatment period.

Another study with baseline levels between 220 and 280 ng/dL reported sustained improvements in sexual function over 36 months, with target testosterone levels maintained in the mid-normal range (500-800 ng/dL), and no significant increase in adverse events when patients were adequately monitored [9]. Similar outcomes have been observed in other long-term studies, which also documented improvements in orgasmic function, sexual confidence, and partner satisfaction [9-11].

Overall, the evidence indicates that TRT is effective in restoring sexual vitality in men aged 50 years and above with confirmed testosterone deficiency, especially when baseline levels are <300 ng/dL and treatment is titrated to achieve mid-normal physiological concentrations.

Testosterone is a potent anabolic hormone that regulates muscle protein synthesis, inhibits proteolysis, and modulates body fat distribution through androgen receptor-mediated pathways in skeletal muscle and adipose tissue [3,12]. In aging men, particularly when serum testosterone levels drop below 300 ng/dL, there is an increased prevalence of sarcopenia, reduced muscle strength, and accumulation of visceral adiposity [1,8,13]. These unfavorable changes contribute to decreased mobility, higher metabolic risk, and overall decline in functional independence [2,3].

RCTs consistently show that TRT in hypogonadal men aged 50 years and older leads to significant improvements in body composition. In RCT (mean baseline testosterone ~234 ng/dL), one year of transdermal TRT increased lean body mass by an average of 1.62 kg and reduced fat mass by 1.45 kg compared with placebo, with most changes observed in the first six months [4,14]. Functional outcomes also improved, with modest but significant gains in leg-press strength and stair-climbing power [4,14].

Other large-scale studies demonstrated sustained increases in lean mass and reductions in waist circumference over up to five years of treatment, with serum testosterone maintained between 500 and 800 ng/dL [9,15]. A meta-analysis confirmed that TRT improves muscle mass and maximal strength, especially in men with baseline testosterone <250 ng/dL [16]. Furthermore, another study reported that intramuscular formulations may produce slightly greater increases in lean body mass than transdermal applications, though both are effective [4,17].

The anabolic effects of TRT are further enhanced when combined with resistance training, as shown by combined-intervention trials in older hypogonadal men, which demonstrated additive benefits on muscle strength, gait speed, and overall physical performance [12,18]. Given the role of muscle preservation in preventing disability and frailty, TRT represents a valuable therapeutic tool to support healthy aging in appropriately selected men.

Testosterone plays a crucial role in skeletal health by stimulating osteoblast activity, inhibiting osteoclast-mediated bone resorption, and enhancing calcium retention through direct androgen receptor signaling and aromatization to estradiol [3,19]. Declining testosterone levels in men aged 50 years and above, particularly below 300 ng/dL, are associated with reduced bone mineral density (BMD), deterioration of trabecular microarchitecture, and increased fracture risk [1,8,20]. The prevalence of osteoporosis in hypogonadal men over 65 years is estimated at 15-20%, a figure that rises in those with comorbidities such as type 2 diabetes and chronic glucocorticoid use [20].

RCTs have demonstrated that TRT significantly increases BMD, especially at the lumbar spine and femoral neck, in men with low baseline testosterone [4,10,21]. In one RCT, one year of transdermal TRT in men with baseline testosterone around 234 ng/dL led to a mean increase in lumbar spine volumetric BMD of 7.5% and in hip BMD of 3.3%, along with improvements in estimated bone strength compared to placebo [4,21]. These benefits were more pronounced in men achieving serum testosterone levels in the mid-normal range (500-800 ng/dL) [4,21].

Long-term data also suggest sustained skeletal benefits. Extension studies have reported continued gains in BMD over three years of therapy, with a plateau effect thereafter [4,22]. Meta-analyses confirm that TRT improves BMD and bone strength, with a more robust effect at the spine than at the hip, and the potential to reduce fracture risk when therapy is maintained for more than two years [10,23].

In clinical practice, TRT should be considered a key component in the prevention and treatment of osteoporosis in hypogonadal men, especially when combined with adequate vitamin D and calcium intake, weight-bearing exercise, and fall prevention strategies [19,23].

Metabolic health and cardiovascular profile

Testosterone deficiency in men aged 50 years and above is associated with increased visceral adiposity, insulin resistance, dyslipidemia, and a higher prevalence of metabolic syndrome [1,3,13]. Low testosterone levels (<300 ng/dL) have been independently linked to type 2 diabetes, central obesity, and adverse cardiovascular outcomes [1,8,24]. Restoration of physiological testosterone concentrations through TRT has shown favorable effects on multiple metabolic parameters, contributing to improved cardiometabolic health.

RCTs and observational studies indicate that TRT in hypogonadal men significantly reduces fasting glucose, hemoglobin A1c, and insulin resistance as measured by HOMA-IR [15,25,26]. Improvements in lipid profiles have also been reported, with consistent reductions in total cholesterol and triglycerides, modest decreases in LDL cholesterol, and variable effects on HDL cholesterol [15,25].

Long-term registry data demonstrate that sustained TRT (testosterone levels maintained between 500 and 800 ng/dL) in hypogonadal men leads to progressive reductions in waist circumference, BMI, and markers of systemic inflammation such as C-reactive protein [15,27]. These findings are supported by meta-analyses showing that TRT lowers components of the metabolic syndrome and may delay or prevent the onset of type 2 diabetes in predisposed men [16,25,26].

Regarding cardiovascular outcomes, contemporary evidence challenges earlier concerns about TRT increasing cardiovascular risk. Large prospective cohort studies and meta-analyses have found no increase in major adverse cardiovascular events (MACE) when TRT is prescribed according to guidelines and with proper monitoring [24,25,28]. Some studies even suggest a protective effect through improved endothelial function, increased arterial compliance, and a reduction in pro-inflammatory markers [24,28].

Collectively, the evidence supports the role of TRT as a beneficial adjunct in managing metabolic health and potentially mitigating cardiovascular risk in appropriately selected men with testosterone deficiency, provided treatment is individualized and closely monitored.

Testosterone receptors are widely distributed in brain regions involved in mood regulation, motivation, and cognition, including the amygdala, hippocampus, and prefrontal cortex [3,29]. Declining testosterone levels (<300 ng/dL) in aging men have been associated with depressive symptoms, reduced motivation, cognitive slowing, and diminished overall vitality [1,8,30]. The prevalence of mild depressive symptoms in hypogonadal men over 50 can reach 20-30%, often coexisting with fatigue and decreased quality of life [29,30].

RCTs indicate that TRT can improve mood, energy, and general well-being in men with confirmed testosterone deficiency. In one RCT, men with baseline testosterone around 230-250 ng/dL reported significant improvements in mood scores, energy levels, and self-perceived vitality after 12 months of transdermal TRT [4,31]. Cognitive testing in the same cohort showed modest but significant improvements in verbal memory and executive function, particularly in those with the lowest baseline cognitive performance [31].

Long-term observational data suggest that maintaining testosterone levels in the mid-normal range (500-800 ng/dL) is associated with sustained mood benefits and reduced risk of incident depressive episodes [27,32]. Additionally, neuroimaging studies have reported increased functional connectivity in prefrontal cortical networks and improved cerebral perfusion following TRT, suggesting potential neuroprotective effects [29,33].

Quality-of-life measures, assessed through validated questionnaires such as the Aging Males' Symptoms (AMS) scale and the Short Form-36 (SF-36), consistently show improvements in physical functioning, social engagement, and emotional well-being after 6-12 months of therapy [6,27,34]. These benefits are most evident when TRT is integrated into a multidisciplinary approach that addresses sleep quality, nutrition, and physical activity.

Overall, the evidence supports TRT as a valuable intervention for enhancing mood, preserving cognitive performance, and improving quality of life in hypogonadal men aged 50 and above, with effects that extend beyond purely physical health outcomes.

Safety considerations

Safety remains a central consideration in TRT, particularly in older men, where historical concerns have focused on cardiovascular events, prostate health, and erythrocytosis [3,5,35]. Early observational studies raised concerns about a potential association between TRT and increased cardiovascular risk; however, these findings were limited by methodological flaws, confounding factors, and heterogeneous patient populations [35,36].

Recent high-quality evidence indicates that when TRT is prescribed according to established guidelines, with appropriate dosing and monitoring, the risk of MACE is not increased [24,25,28,37]. In fact, some studies report improved cardiovascular function, endothelial health, and reduced all-cause mortality among hypogonadal men whose testosterone levels are restored to the mid-normal range (500-800 ng/dL) [24,28,37].

Prostate health remains another critical safety domain. Current data demonstrate that TRT in men without active prostate cancer does not increase the incidence of prostate cancer or accelerate its progression [38,39]. Small, transient increases in prostate-specific antigen (PSA) may occur during the first year of therapy, but these typically stabilize and remain within normal limits [39]. Clinical guidelines recommend baseline PSA and digital rectal examination before initiating TRT, followed by periodic reassessment [2,3,5].

Erythrocytosis is the most common dose-related adverse effect of TRT, particularly with injectable formulations [17,40]. Monitoring of hematocrit levels every three to six months during the first year is recommended, with dose adjustments or temporary discontinuation if hematocrit exceeds 54% [3,5,17].

Overall, the safety profile of TRT is favorable when therapy is individualized, contraindications are respected, and systematic monitoring is implemented. The benefits in symptom relief, physical function, and quality of life often outweigh the manageable risks in appropriately selected men with testosterone deficiency.

A concise summary of the clinical benefits, risks, and recommended monitoring parameters for TRT in men aged 50 years and above is presented in Table 1.

**Table 1:** Summary of Benefits, Risks, and Monitoring Parameters of Testosterone Replacement Therapy (TRT) in Men Aged 50 Years and Above Abbreviations: TRT, testosterone replacement therapy; BMD, bone mineral density; QoL, quality of life; AMS, Aging Males’ Symptoms scale; SF-36, Short Form-36 questionnaire; PSA, prostate-specific antigen; DRE, digital rectal examination; Hct, hematocrit; Hgb, hemoglobin; MACE, major adverse cardiovascular events; IM, intramuscular.

Domain	Evidence-Based Benefits	Potential Risks/Adverse Effects	Recommended Monitoring	Representative References
Sexual function	↑ Libido, ↑ erectile function, ↑ sexual satisfaction, improved orgasmic function and partner satisfaction	Variable response; multifactorial erectile dysfunction may persist	Serum testosterone (baseline, 3–6 mo); validated questionnaires for sexual function	[4,6–11]
Musculoskeletal	↑ Lean body mass, ↓ fat mass, ↑ strength (leg press, stair climb), improved physical performance; additive with exercise	Fluid retention/edema, exacerbation of sleep apnea; mild formulation-dependent differences (IM > transdermal)	DXA in high-risk patients; physical function measures; periodic assessment of strength	[4,12–18]
Bone health	↑ BMD (lumbar spine, hip), improved trabecular architecture, reduced fracture risk with long-term therapy	Benefits plateau after ~3 years; inconsistent fracture prevention data	DXA every 1–2 years in osteopenic/osteoporotic men; ensure vitamin D, calcium, and exercise	[4,10,19–23]
Metabolic profile	↓ Visceral adiposity, ↓ waist circumference, ↑ insulin sensitivity, ↓ fasting glucose and HbA1c, improved lipids	Variable HDL response; long-term CV outcome data heterogeneous	Fasting glucose, HbA1c, lipid panel (baseline, 6–12 mo); waist circumference/BMI	[1,3,8,13,15–16,25–27]
Cardiovascular	Improved angina threshold, possible endothelial/arterial function benefits; no ↑ in MACE with guideline-based TRT	Controversial data from older studies; risk of MACE if misused or unmonitored	Hematocrit (baseline, 3–6 mo, then annually); blood pressure; cardiovascular risk stratification	[24,25,28,35–37]
Mood & cognition	↑ Mood, energy, vitality; improved verbal memory, executive function, QoL (AMS, SF-36); ↓ depressive symptoms	Mood lability, irritability in some patients	QoL questionnaires (AMS, SF-36); cognitive screening in older/at-risk patients	[4,27,29–34]
Prostate health	No ↑ incidence/progression of prostate cancer with proper selection; transient PSA rise possible	Theoretical risk of prostate growth; contraindicated in active prostate cancer	Baseline DRE and PSA; repeat at 3–12 mo, then annually	[2,3,5,38–39]
Hematology	TRT stimulates erythropoiesis (↑ Hgb, Hct)	Erythrocytosis (>54% Hct) is the most common dose-related AE, esp. with injectables	Hgb/Hct baseline, 3–6 mo, then annually; adjust dose or pause TRT if Hct >54%	[3,5,17,40]

Limitations and future directions

Although the body of evidence on TRT in men aged 50 years and above has expanded substantially, several limitations in the current literature warrant consideration. First, many RCTs have relatively small sample sizes, short durations of follow-up (often ≤12 months), and may not fully capture long-term outcomes such as fracture prevention, cardiovascular events, and cancer incidence [4,21,23,37]. Additionally, variability in inclusion criteria, baseline testosterone thresholds, and outcome measures complicates direct comparisons across studies [2,3,6].

Another limitation is the underrepresentation of certain patient subgroups, such as men with multiple comorbidities, those from diverse ethnic backgrounds, and individuals over the age of 75 [1,2,27]. Moreover, while meta-analyses provide pooled estimates of TRT effects, heterogeneity in study design, formulations (intramuscular, transdermal, oral), and monitoring protocols introduces uncertainty into the interpretation of results [6,16,25].

From a methodological perspective, the absence of standardized definitions for LOH and consistent biochemical cutoffs across guidelines hinders the development of universally applicable clinical recommendations [2,3,5].

Future research should prioritize large-scale, multicenter RCTs with longer follow-up to clarify the long-term safety profile of TRT, particularly regarding cardiovascular health, prostate outcomes, and fracture reduction [23,37,38]. Studies incorporating advanced imaging, molecular biomarkers, and patient-reported outcomes could provide deeper insights into the mechanisms and full spectrum of TRT benefits. Furthermore, trials assessing combined interventions, such as TRT with resistance exercise, nutritional optimization, or pharmacological agents targeting bone and muscle, may reveal synergistic effects in promoting healthy aging [12,18,19].

Ultimately, a more individualized approach to TRT, guided by precision medicine tools and patient-specific risk-benefit profiles, is likely to optimize outcomes while maintaining safety. Table 1 summarizes the main physiological domains impacted by TRT in men aged 50 years and above, integrating evidence from RCTs, long-term observational studies, and meta-analyses. This table consolidates the current literature on benefits, safety considerations, and representative references for each clinical domain, serving as a concise evidence-based guide for clinical decision-making.

## Conclusions

In men aged 50 years and above with confirmed testosterone deficiency, TRT provides consistent and clinically meaningful benefits across sexual, musculoskeletal, metabolic, cardiovascular, and neuropsychological domains when prescribed according to evidence-based guidelines. These effects vary with baseline androgen status, treatment formulation, duration, and comorbidities, highlighting the importance of individualized strategies. With appropriate patient selection and structured monitoring, TRT shows a favorable safety profile, with manageable risks such as erythrocytosis and minimal impact on prostate health or cardiovascular events.

Despite these advances, significant gaps remain. Future large-scale RCTs with standardized definitions, diverse populations, longer follow-up, and functional outcomes are needed to refine current recommendations. Until then, judicious use of TRT, tailored to patient needs and combined with lifestyle optimization, represents a safe and effective approach to improve quality of life and functional capacity in older men with androgen deficiency.
